# Correlation between Iliac Tilt and traditional sagittal parameters in adolescents

**DOI:** 10.1186/s13018-025-05642-1

**Published:** 2025-03-01

**Authors:** ZengHui Zhao, Hao Qi, Anqi Zhao, Hongru Yuan, Peng Zhang, Chenchen Wang, Chenxi Wang, Di Zhang

**Affiliations:** 1 Department of Spine Surgery, the Third Hospital of Hebei Medical University, Shijiazhuang, 050051 China; 2 Department of Biochemistry and Molecular Biology, Key Laboratory of Neural and Vascular Biology, Ministry of Education, Hebei Medical University, Shijiazhuang, 050017 China

**Keywords:** Iliac Tilt, Pelvic Tilt, Pubic-Sacral angle, Sagittal balance, Risser staging

## Abstract

**Objective:**

This study aims to systematically validate the reliability and applicability of Iliac Tilt (IT) as a parameter for sagittal balance assessment, explore its relationship with traditional parameters such as Pelvic Tilt (PT) and Pubic-Sacral Angle (PSA), and analyze its performance across different stages of skeletal development.

**Methods:**

A retrospective study was conducted with 118 healthy pediatric participants. Full-length sagittal radiographs were obtained using the advanced EOS imaging system, and relevant sagittal plane parameters were measured using standardized methods. Participants were grouped based on Risser staging, and statistical analyses, including one-way analysis of variance (ANOVA), Pearson correlation analysis, and linear regression, were performed.

**Results:**

Iliac Tilt (IT) was significantly negatively correlated with Pelvic Tilt (PT) (*r* = -0.761, *p* < 0.001) and significantly positively correlated with Pubic-Sacral Angle (PSA) (*r* = 0.814, *p* < 0.001). Linear regression analysis revealed that IT was a significant predictor of both PT and PSA, with R² values of 0.736 and 0.717, respectively. Additionally, across different Risser stages, IT demonstrated high stability, while PSA and T1 Slope-Pelvic Incidence (T1SPI) showed significant changes with staging.

**Conclusion:**

As a novel parameter, Iliac Tilt (IT) demonstrates high reliability and broad applicability in reflecting posterior pelvic tilt and sagittal spinal balance. Compared to traditional parameters, IT offers a simpler measurement process, making it suitable for complex cases with limited imaging quality. It provides an efficient evaluation tool for clinical and research applications.

## Highlights

**Introduction of a Novel Parameter**: This study systematically validates Iliac Tilt (IT) as a new and reliable parameter for assessing sagittal spinal balance in adolescents, demonstrating its broad applicability.

**Strong Correlations Identified**: IT shows a significant negative correlation with Pelvic Tilt (PT) (r = -0.761) and a significant positive correlation with Pubic-Sacral Angle (PSA) (r = 0.814), highlighting its effectiveness in reflecting pelvic and spinal alignment.

**Stability Across Developmental Stages**: IT remains highly stable across different Risser stages of skeletal development, unlike traditional parameters such as PSA and T1 Slope-Pelvic Incidence (T1SPI), which vary significantly with skeletal maturity.

**Clinical Advantages**: Compared to traditional sagittal parameters, IT offers a simpler and more reliable measurement process by requiring only unilateral iliac imaging, making it suitable for complex cases with limited imaging quality or special anatomical structures.

**Foundation for Future Research**: The findings establish IT as a valuable tool for clinical and research applications in spinal health assessment, recommending further studies to explore its applicability in diverse age groups and pathological conditions.

## Introduction

Sagittal spinal balance is a critical indicator for maintaining normal posture and function in the human body. Its assessment holds significant clinical relevance in scenarios such as scoliosis, degenerative spinal diseases [1], and postoperative rehabilitation management following spinal surgery [2]. In the evaluation of sagittal balance, the Pelvic Tilt (PT) is widely utilized as a core parameter to assess the degree of posterior pelvic tilt [3–4]. However, the measurement of PT is highly dependent on imaging techniques, particularly requiring clear visualization of both femoral heads. This technical requirement can be challenging to meet in certain complex cases, such as patients with lumbosacral transitional vertebrae or severe hip joint deformities [5]. In these situations, the accuracy and feasibility of PT measurements are significantly compromised, thereby affecting their clinical applicability and reliability.

To address these limitations, researchers have progressively sought to introduce alternative parameters to enhance the operability and universality of sagittal balance assessments [6]. In this context, the Iliac Tilt (IT) has emerged as a novel parameter garnering widespread attention [7]. IT reflects the degree of posterior pelvic tilt by analyzing the angle of the iliac cortical density line, with a relatively straightforward measurement process that requires only unilateral iliac imaging. This makes IT particularly suitable for patients with limited image quality or special anatomical structures. As a potential alternative to PT, IT has demonstrated good correlation and practicality in some studies. However, existing research primarily focuses on simple correlation analyses between IT and PT, lacking a systematic exploration of IT in relation to other sagittal plane parameters and insufficient investigation of IT performance across different stages of skeletal development [8–9]. These limitations hinder the further promotion and application of IT.

Therefore, this study aims to address these gaps by systematically validating the reliability and applicability of IT as a parameter for sagittal balance assessment. We have designed a rigorous study to explore the relationship between IT and traditional sagittal plane parameters (such as PT and Pubic-Sacral Angle [PSA]) and to analyze the performance of IT across different stages of skeletal development. Utilizing the advanced EOS imaging system, this study ensures data accuracy and consistency through standardized measurement and analysis methods. Additionally, subjects are grouped based on Risser stages to more precisely elucidate the impact of skeletal development on IT and related parameters.

The objective of this study extends beyond validating the feasibility of IT as an alternative to PT. It also seeks to uncover the multidimensional application potential of IT in the assessment of sagittal spinal balance. Through systematic analysis of IT in relation to traditional parameters and an in-depth exploration of its applicability, we aim to provide a more convenient and efficient evaluation tool for clinical practice and research. This will offer new perspectives and methodologies for the management of spinal health and the diagnosis, treatment, and prognostic evaluation of related spinal disorders.

## Materials and methods

### Study design

The study protocol was approved by the Ethics Committee of the Third Hospital of Hebei Medical University, and informed consent was obtained from all patients. All methods were conducted in accordance with relevant guidelines and regulations of our institution.

This retrospective study analyzed 118 pediatric patients (53 females and 65 males) who underwent full-length anteroposterior and lateral radiographs of the spine at our hospital. The participants included in the study were those who underwent spinal X-rays for scoliosis screening during outpatient visits, but were found to have no scoliosis upon screening.

Exclusion criteria: (1) Spinal deformities, including scoliosis and congenital spinal anomalies (e.g., Scheuermann’s disease). (2) History of spinal surgery, trauma, or spinal disease. (3) Hip joint disorders such as developmental dysplasia of the hip, hip arthritis, femoral head necrosis, or prior total hip arthroplasty. (4) Personal history of malignancy. (5) Incomplete or poor-quality imaging data that could compromise measurement accuracy. (6) Patients who did not provide signed informed consent for participation in the study.

### Radiographic image acquisition and parameter measurement

All images were acquired using a low-dose biplanar X-ray system (EOS imaging system: Paris, France). This system allows for simultaneous capture of full-body biplanar images without distortion while reducing radiation exposure compared to conventional full-length X-rays. The subjects underwent EOS imaging in an upright standing position. They were instructed to stand comfortably without leaning, maintain a horizontal gaze, and place their fists on their clavicles during imaging, following previously reported protocols. The images were captured by trained radiologic technicians with over three years of experience operating the system.

**Fig. 1:**
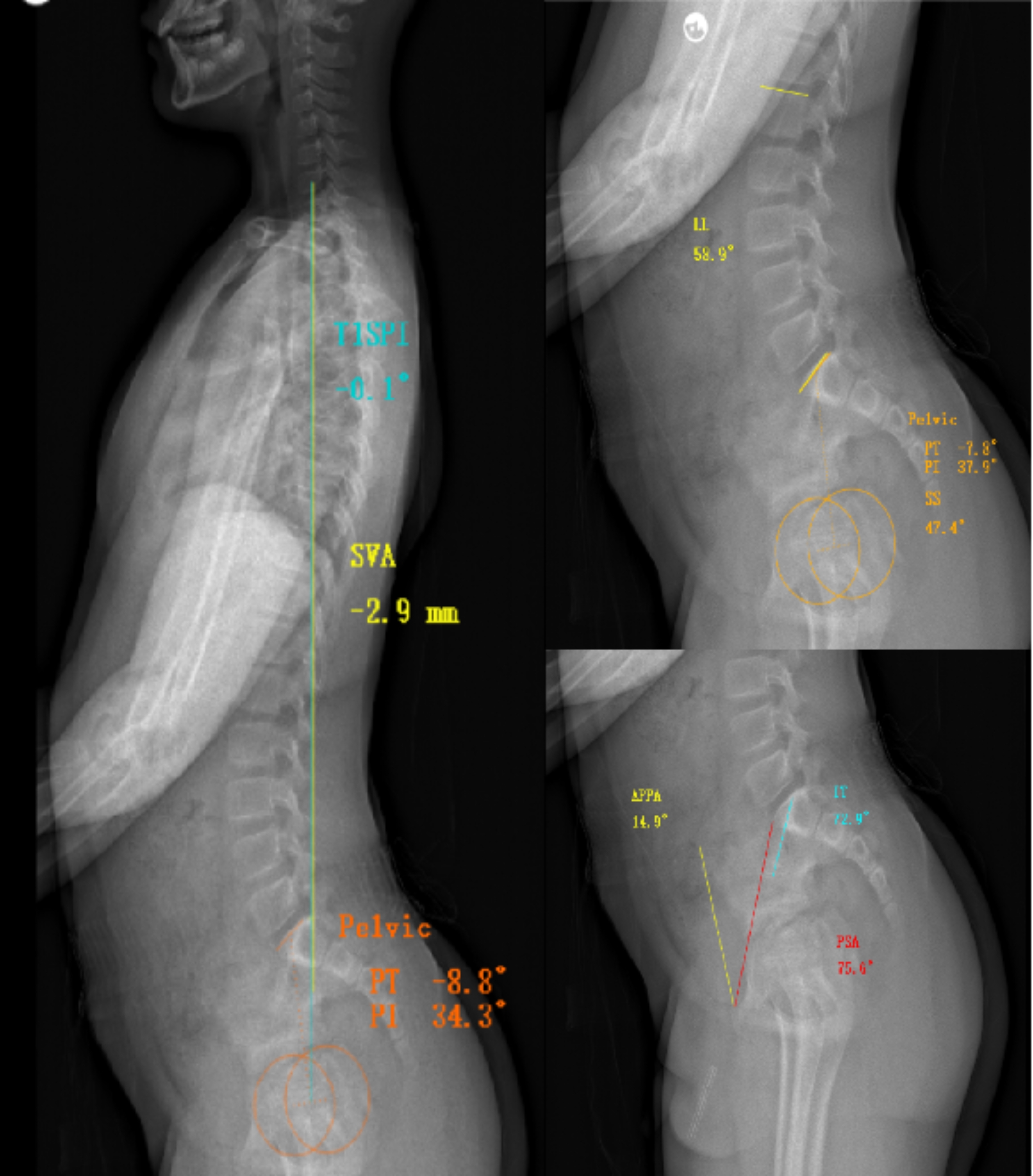
Representation of the measurement of individual spine sagittal parameters in the SurgiMap software

Subsequently, the following sagittal spine-pelvic parameters were measured using Surgimap software (Nemaris Inc., NY, USA, version 1.2.1.82), which is shown in Fig. 1:

IT (Iliac Tilt): IT is defined as the angle between the iliac cortical density line and the horizontal line. Anatomically, there are left and right cortical density lines. However, the intensity of the line depends on the X-ray incidence angle, with the iliac cortical density line farther from the X-ray plate appearing thicker than the nearer one. In many cases, only the farther iliac cortical density line is detectable. When both lines are visible, the nearer one is detected as the upper line, and the farther one as the lower line.

PI (Pelvic Incidence): The angle between a line perpendicular to the midpoint of the S1 superior endplate and the line connecting this point to the midpoint of the femoral head axis.

PT (Pelvic Tilt): The angle between the line connecting the midpoint of the femoral head axis to the midpoint of the S1 superior endplate and the vertical line.

SS (Sacral Slope): The angle between the superior border of the sacrum and the horizontal plane.

LL (Lumbar Lordosis): The angle between the superior endplate of the first lumbar vertebra (L1) and the superior border of the S1 vertebra.

SVA (Sagittal Vertical Axis): The horizontal distance between the vertical line passing through the center of the C7 vertebral body and the posterior border of the S1 superior endplate.

PSA (Pubic-Sacral Angle): The angle between the line connecting the anterior border of the pubis and the anterior border of the S1 superior endplate and the horizontal plane.

APPA (Anterior Pelvic Plane Angle): The angle between the line connecting the bilateral anterior superior iliac spines and the upper edge of the pubic symphysis and the vertical line.

T1SPI (T1 Slope Pelvic Incidence): The angle between the line connecting the center of the T1 vertebral body to the midpoint of the line connecting both femoral heads and the vertical line.

### Interobserver and intraobserver reliability

Three spine surgeons (Observer 1, Observer 2, and Observer 3), all familiar with the Surgimap operation procedures, were instructed on how to measure IT and other parameters using the computer monitor. Measurements were performed twice on two separate occasions, with an interval of four weeks between sessions. Intraobserver consistency was evaluated using intraclass correlation coefficients (ICC).

### Statistical analysis

In this study, descriptive statistical analyses were performed for all relevant variables. Differences between gender and Risser staging groups were compared using one-way analysis of variance (ANOVA) followed by post hoc tests, while independent sample t-tests were employed to assess gender-specific differences. Pearson correlation coefficients were used to evaluate the relationships between variables. Intraclass correlation coefficients (ICC) were calculated to verify the consistency of measurements. All statistical tests were conducted using SPSS software (IBM SPSS Statistics 27.0), with the significance level set at *p* < 0.05.

## Results

### Descriptive statistics and demographics

A total of 118 pediatric patients were included in this study, comprising 53 males (45%) and 65 females (55%). The mean age of the participants was 11.45 years (SD: 2.58; range: 5–15 years). Descriptive statistics revealed that the mean Iliac Tilt (IT) was 67.96° (SD: 7.22; range: 49.6°–87.4°), and the mean Pelvic Incidence (PI) was 42.18° (SD: 8.06; range: 24.3°–64.5°). The Sagittal Vertical Axis (SVA) ranged from − 81.4 mm to 66.4 mm, with a mean of -0.78 mm (SD: 24.23). Detailed data are presented in Table 1.

**Table 1:** Descriptive statistics and demographics

Characteristics		Number		Percentage
**Gender**	Male	53		45%
	Female	65		55%
	Mean	SD	Minimum	Maximum
Age(y)	11.45	2.58	5	15
IT(°)	67.96	7.22	49.6	87.4
PI(°)	42.18	8.06	24.3	64.5
PT(°)	8.23	7.57	-13.8	23.5
SS(°)	34.20	8.59	11.8	53.4
LL(°)	47.27	12.61	16.9	83.6
PSA(°)	67.85	7.39	52.3	86.6
APPA(°)	-5.06	7.09	-20.5	22.6
T1SPI(°)	3.85	2.79	-3.9	14.9
SVA(°)	-0.78	24.23	-81.4	66.4

IT (°): Iliac Tilt. PI (°): Pelvic Incidence. PT (°): Pelvic Tilt. SS (°): Sacral Slope. LL (°): Lumbar Lordosis. PSA (°): Pubic-Sacral Angle. APPA (°): Anterior Pelvic Plane Angle. T1SPI (°): T1 Slope Pelvic Incidence. SVA (°): Sagittal Vertical Axis (please refer to the text for the specific unit used in this study)

### Comparative analysis across Risser stages and gender

One-way ANOVA indicated significant differences in age across Risser staging groups (*p* < 0.001). Post hoc comparisons showed that group A was significantly younger than both group B and group C (*p* < 0.05), consistent across both genders. Within Risser staging groups, significant differences in Pubic-Sacral Angle (PSA) were observed: males in group A had significantly higher PSA values than those in group C (*p* = 0.048), and females in group A had higher PSA values than those in group B (*p* = 0.024). Additionally, T1 Slope Pelvic Incidence (T1SPI) showed significant differences across Risser staging groups in females (*p* = 0.011). However, there were no statistically significant differences in IT, PI, or PT across Risser groups for either gender. Detailed results are shown in Table 2.

Independent sample t-tests revealed no significant gender differences in most parameters, except for Pelvic Tilt (PT) and PSA. Females in Risser stage B had significantly higher PT values compared to males (*p* = 0.038), and females in stage A had higher PSA values than males (*p* < 0.05).

**Table 2:** Comparative analysis of Risser stages across genders for various variables

	A(Risser0 ~ 1)	B(Risser2 ~ 3)	C(Risser4 ~ 5)	ANOVA *P*-value	Post-hoc Comparisons
**Age**(y)					
Male	9.54 ± 1.95	13.00 ± 1.05	13.73 ± 1.16	< 0.001*	A < B,A < C
Female	8.38 ± 2.01	12.95 ± 1.03	13.04 ± 1.33	< 0.001*	A < B,A < C
*P*-value	0.050*	0.899	0.094		
**IT(°)**					
Male	69.27 ± 8.57	68.27 ± 6.62	64.42 ± 5.54	0.133	
Female	70.02 ± 8.04	65.56 ± 4.20	68.58] ± 7.29	0.119	
*P*-value	0.756	0.262	0.050*		
**PI(°)**					
Male	41.67 ± 7.32	41.56 ± 9.20	45.86 ± 7.63	0.215	
Female	39.46 ± 6.08	41.89 ± 8.90	43.31 ± 9.17	0.290	
*P*-value	0.254	0.926	0.349		
**PT(°)**					
Male	6.51 ± 8.49	7.97 ± 7.02	11.78 ± 5.54	0.100	
Female	5.24 ± 7.99	11.26 ± 5.85	8.32 ± 7.55	0.038*	A < B
*P*-value	0.593	0.223	0.104		
**SS(°)**					
Male	35.14 ± 8.78	33.60 ± 8.31	34.74 ± 8.05	0.886	
Female	35.16 ± 7.66	30.62 ± 6.64	34.99 ± 10.68	0.182	
*P*-value	0.995	0.343	0.934		
**LL(°)**					
Male	50.12 ± 11.19	45.88 ± 8.74	45.98 ± 11.17	0.378	
Female	49.02 ± 12.49	42.83 ± 9.58	47.34 ± 17.45	0.359	
*P*-value	0.753	0.399	0.766		
**PSA(°)**					
Male	70.12 ± 8.42	67.19 ± 7.74	63.96 ± 5.66	0.048*	A < C
Female	70.64 ± 7.75	64.70 ± 5.49	67.95 ± 6.49	0.024*	A > B
*P*-value	0.823	0.383	0.050		
**APPA(°)**					
Male	-6.63 ± 7.73	-5.44 ± 5.27	-4.74 ± 5.76	0.673	
Female	-4.24 ± 8.24	-2.37 ± 5.61	-6.08 ± 7.57	0.254	
*P*-value	0.308	0.162	0.533		
**T1SPI(°)**					
Male	4.18 ± 2.99	2.84 ± 1.61	3.22 ± 2.25	0.283	
Female	3.56 ± 3.11	5.08 ± 2.79	3.58 ± 2.80	0.173	
*P*-value	0.485	0.011*	0.652		
**SVA(°)**					
Male	-4.92 ± 23.87	10.36 ± 21.98	8.47 ± 22.80	0.095	
Female	-4.29 ± 21.43	-9.52 ± 24.72	3.41 ± 26.11	0.213	
*P*-value	0.923	0.038*	0.525		

For the ANOVA *P*-values, < 0.05 is considered significant.

For the independent samples t-test based on gender grouping, *p*-value < 0.5 is considered significant.

IT (°): Iliac Tilt. PI (°): Pelvic Incidence. PT (°): Pelvic Tilt. SS (°): Sacral Slope. LL (°): Lumbar Lordosis. PSA (°): Pubic-Sacral Angle. APPA (°): Anterior Pelvic Plane Angle. T1SPI (°): T1 Slope Pelvic Incidence. SVA (°): Sagittal Vertical Axis (please refer to the text for the specific unit used in this study)

### Correlation analysis

**Fig. 2:**
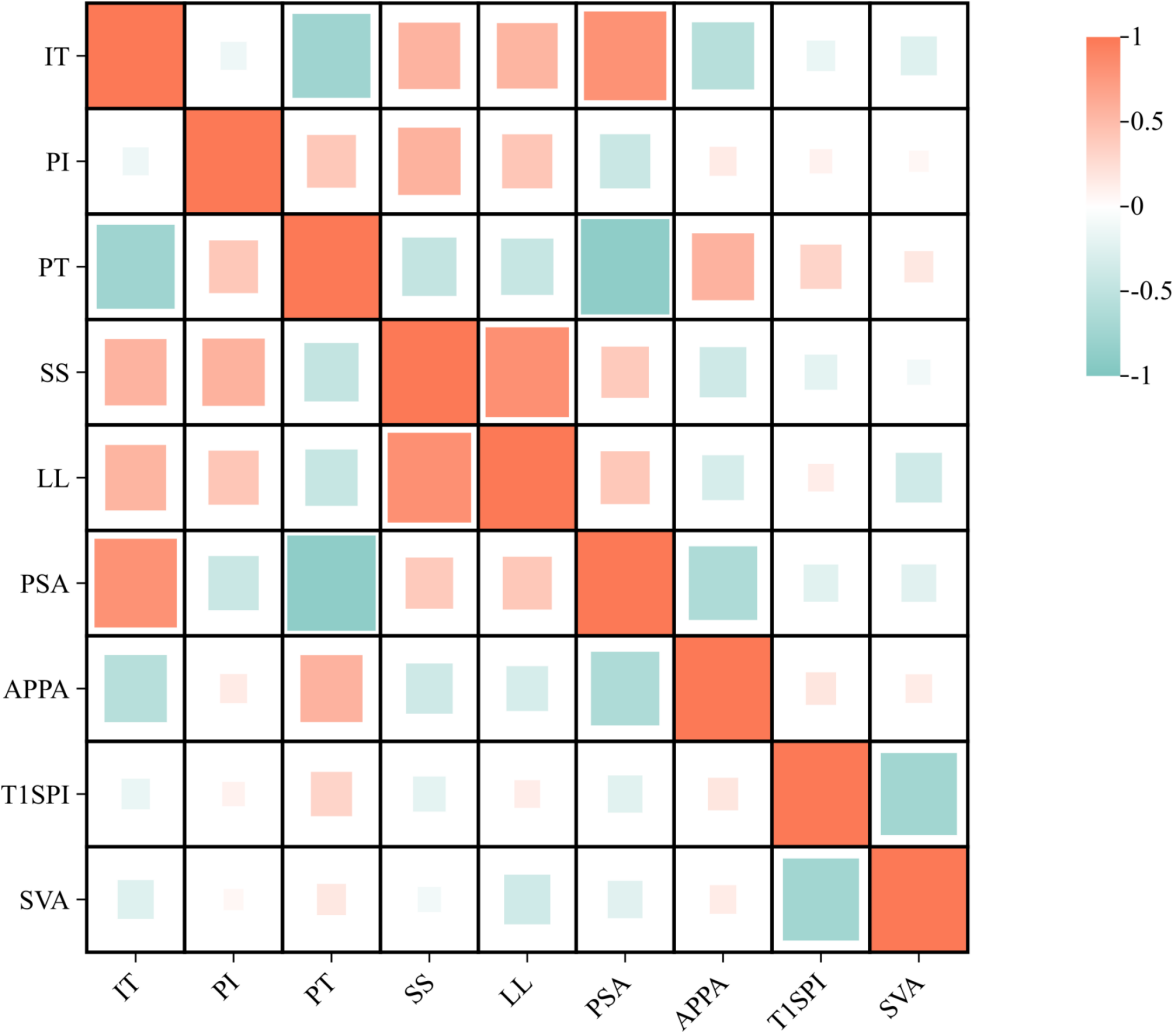
Heat map generated using Pearson correlation analysis. The color gradient ranges from red (positive correlation) to blue (negative correlation), where higher or lower values indicate stronger correlation. Asterisks (*) in the figure denote statistically significant differences (*p* < 0.05)

Pearson correlation analysis identified significant relationships between sagittal spine-pelvic parameters (Table 3). IT was strongly and positively correlated with PSA (*r* = 0.814, *p* < 0.001) but negatively correlated with PT (*r* = -0.761, *p* < 0.001). PI showed moderate positive correlations with Sacral Slope (SS) (*r* = 0.575, *p* < 0.001) and PT (*r* = 0.413, *p* < 0.001). Additionally, SVA and T1SPI exhibited a negative correlation (*r* = -0.739, *p* < 0.001). The correlation heatmap is shown in Fig. 2.

**Table 3:** Correlation analysis of sagittal spinal parameters

	IT	PI	PT	SS	LL	PSA	APPA	T1SPI	SVA
IT	1	-0.136	-0.761**	0.565**	0.555**	0.814**	-0.573**	− 0.165	− 0.258**
PI		1	0.413**	0.575**	0.428**	-0.429**	0.149	0.096	0.061
PT			1	-0.476**	-0.453**	-0.887**	0.571**	0.319**	0.173
SS				1	0.826**	0.396**	-0.385**	-0.216*	-0.104
LL					1	0.414**	-0.325**	0.133	-0.379**
PSA						1	-0.646**	-0.240**	-0.242**
APPA							1	0.188*	0.144
T1SPI								1	− 0.739**
SVA									1

Pearson correlation coefficients are shown. A single asterisk (*) indicates *p* < 0.05, and a double asterisk (**) indicates *p* < 0.01

### Regression analysis

**Fig. 3:**
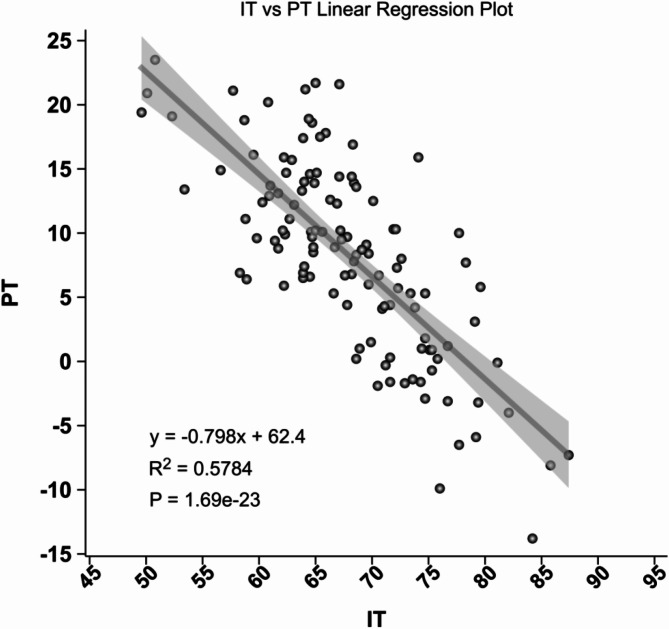
Linear regression analysis showing the relationship between Iliac Tilt (IT) and Pelvic Tilt (PT)

**Fig. 4:**
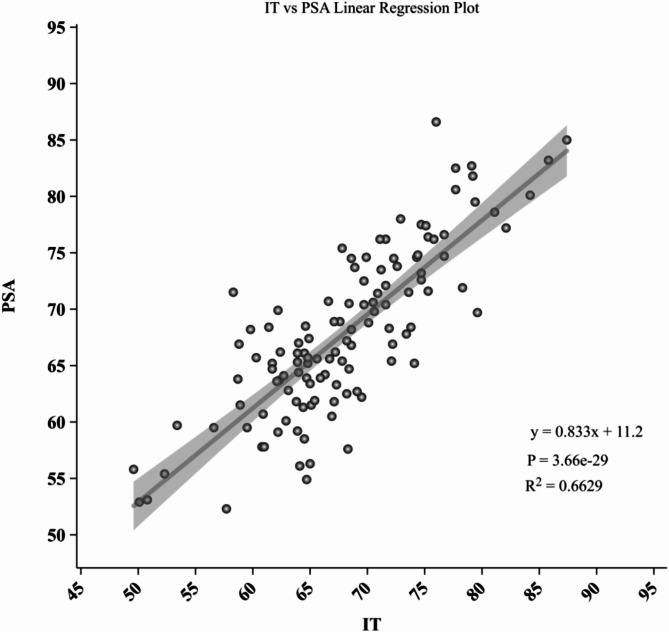
Scatter plot illustrating the significant positive correlation between Iliac Tilt (IT) and Pubic-Sacral Angle (PSA)

Linear regression analysis demonstrated that IT significantly predicted both PT and PSA. The regression equation for PT was PT = -0.7302 * IT − 0.0183, with an R² value of 0.736 (*p* < 0.001). For PSA, the regression equation was PSA = 0.8184 * IT − 0.0553, with an R² value of 0.717 (*p* < 0.001). The regression model parameters are detailed in Table 4. The linear regression graphs are shown in Figs. 3 and 4.

**Table 4:** Linear relationship parameters between IT, PT, and PSA

Predictive Variable	Item	PT	PSA
IT	Regression Coefficient	-0.7302	0.8184
	Intercept	-0.0183	-0.0553
	R² Value	0.736	0.717
	Adjusted R² Value	0.733	0.714
	(Prob (F-statistic))	2.57e-35	1.44e-33
	*p*-value	< 0.001	< 0.001
	Prediction Equation	PT = -0.7302 * IT − 0.0183	PSA = 0.8184 * IT − 0.0553

### Measurement reliability

Interobserver and intraobserver reliability assessments, evaluated using intraclass correlation coefficients (ICC), showed excellent consistency. For IT, the ICC for single measurements was 0.90, and for average measurements, it was 0.96. Similarly, PT had ICC values ranging from 0.88 to 0.94, and PSA ranged from 0.87 to 0.93. These results indicate high reliability across observers and measurement occasions. Detailed ICC values are provided in Table 5.

**Table 5:** Intraclass correlation coefficient (ICC)

Measurement Parameter	ICC Value (Single Measurement)	ICC Value (Single Measurement)	ICC Value (Average Measurement)
IT	0.90	0.96	0.88–0.92
PT	0.88	0.94	0.83–0.92
PSA	0.87	0.93	0.82–0.91

The ICC values are reported for both single and average measurements, corresponding to intraobserver and interobserver reliability, respectively. An ICC greater than 0.75 indicates good reliability, while an ICC greater than 0.90 indicates excellent reliability

## Discussion

This study expands the understanding of spinal sagittal balance by introducing the Iliac Tilt (IT) as an innovative parameter, particularly addressing the limitations associated with the traditional Pelvic Tilt (PT) in clinical applications. As early as 2015, Toshio Doi [7] and colleagues proposed that the angle of the cortical density lines of the ilium could serve as an alternative in assessing pelvic and spinal sagittal parameters. Their research highlighted that many patients, due to anatomical or pathological reasons, face challenges in obtaining clear bilateral femoral head imaging through conventional imaging techniques, resulting in inaccurate or unfeasible PT measurements. However, Doi et al.‘s study was limited to a simple correlation analysis between IT and PT, without further exploring the relationship of IT with other key parameters or its broad applicability in diverse clinical scenarios. Building on Doi’s findings, this study employed a more rigorous research design to systematically validate the reliability and applicability of IT, unveiling its potential value in the assessment of spinal sagittal balance.

The innovation of this study lies in the systematic analysis of the correlation between IT and traditional sagittal parameters, as well as their performance across different skeletal development stages. We found a significant negative correlation between IT and PT (*r* = -0.761, *p* < 0.001), confirming IT’s sensitivity and accuracy in reflecting posterior pelvic tilt. This result is consistent with the findings of Smith et al. (2018), who also reported a strong negative correlation between IT and PT in adolescent scoliosis patients. Additionally, IT showed a strong positive correlation with the Pubic-Sacral Angle (PSA) (*r* = 0.814, *p* < 0.001), revealing for the first time the multidimensional applicability of IT as a critical parameter of pelvic anatomical structure. This bidirectional association not only further solidifies IT’s position within the sagittal parameter framework but also provides robust support for its clinical application. As demonstrated by our team member Qi Hao [10] in a previous study involving 217 asymptomatic children, incorporating IT into the study of spinal sagittal balance parameters holds significant importance for the research of sagittal balance. Similarly, Zhang Wen [11] and colleagues incorporated the C7-Sacral Tilt (C7ST) into spinal sagittal balance studies, thereby enriching the parameter assessment system.

Methodologically, we rigorously controlled the selection of study subjects and the measurement of parameters to ensure the utmost accuracy of data and reliability of results. This study included 118 adolescent patients who were asymptomatic or undergoing screening for suspected scoliosis. Full-length spinal lateral images were obtained using the EOS imaging system, with all images captured in an upright posture. Sang Bum Kim [12] has indicated that the EOS system exhibits excellent reliability in assessing spinal and pelvic sagittal lines. Compared to traditional imaging techniques, the EOS system significantly reduces radiation exposure while maintaining high-quality images, providing a safer detection method for this sensitive population [13]. Adolescence is a critical period for spinal development, during which rapid skeletal growth can lead to significant changes in sagittal balance parameters. We used Risser staging as the basis for grouping instead of traditional age-based grouping, thereby avoiding confounding factors introduced by individual differences in developmental speed. Prudence Wing Hang Cheung [14] and colleagues, through indices such as the Proximal Femoral Maturity Index (PFMI), Risser staging, Sanders classification, and distal radius and ulna classification, assessed the risk of progression in adolescent idiopathic scoliosis during different stages of brace treatment. This study provided a reference for our grouping methodology, enabling us to more clearly observe the trends in spinal sagittal balance parameters across different skeletal maturity stages.

The results indicate that Risser staging is closely related to sagittal parameters, particularly showing significant associations with the Pubic-Sacral Angle (PSA) and the T1 Spine Pelvic Incidence (T1SPI). These findings have not been clearly established in previous studies. In the Risser 0–1 group, representing skeletal immaturity, PSA values were significantly higher than those in the partially mature Risser 2–3 group and the fully mature Risser 4–5 group, reflecting the dynamic changes in pelvic and spinal morphology with skeletal development. Furthermore, IT demonstrated stable correlations across all groups, further supporting its potential as a stable parameter across different age stages.

Clinically, the measurement of PT is limited in complex cases due to its dependence on image quality and the visibility of bilateral femoral heads [15]. This poses challenges in patients with sacral transitional vertebrae, severe hip joint deformities, or inadequate imaging conditions [16–17]. In contrast, IT measurement only requires standard lumbar lateral images, making it simpler to perform and highly reproducible. This study confirmed the feasibility of IT as an alternative parameter to PT by validating its strong correlation with PT. Additionally, IT exhibited significant correlation with PSA, providing both theoretical support and practical evidence for its use in broader spinal balance assessments. The application of IT not only reduces the technical barriers for radiological assessments in patients but also effectively decreases economic costs and radiation exposure, thereby enhancing assessment efficiency and patient compliance.

Despite the significant findings of this study, several limitations warrant further investigation. Firstly, Our study focused on a pediatric population without major spinal or hip pathologies, allowing us to establish normative relationships between Iliac Tilt (IT) and other sagittal parameters. Consequently, the generalizability of these findings to adults or patients with significant spinal or hip disorders (e.g., degenerative spine disease, advanced hip osteoarthritis, or post-surgical conditions) may be limited. Future research should explore IT measurements in various pathological conditions and across a broader age range to confirm its clinical utility. Evaluating IT in adult populations with different anatomical or radiographic challenges will help clarify whether it remains a reliable parameter under those circumstances. Future research should expand the sample size to include a broader range of ages and pathological conditions to enhance the generalizability of the results. Secondly, although the EOS imaging system was used to ensure high-quality imaging and consistent measurements, there may be measurement discrepancies between different imaging devices. Therefore, it is necessary to further standardize IT measurement methods and develop uniform operational guidelines to facilitate its application in multicenter studies and clinical practice. Additionally, this study employed a cross-sectional design, lacking longitudinal observation of dynamic changes in IT and related parameters. Future studies should incorporate long-term follow-up to explore the predictive value of IT in the progression of scoliosis and other spinal deformities. Another, the scope of the study could be further expanded to encompass populations of different ethnicities, regions, and age groups, thereby validating the applicability and stability of Iliac Tilt (IT) on a larger scale and in more diverse groups. In addition, prospective research focusing on specific spinal pathologies (such as degenerative lumbar disorders, scoliosis, or postoperative recurrence) may be conducted to assess the role of IT in early diagnosis, treatment monitoring, and prognostic evaluation. By utilizing multi-center, large-sample, and longitudinal follow-up designs, researchers can gain a more comprehensive understanding of the relationship between IT and other sagittal parameters, as well as its clinical significance, ultimately enhancing its role in the diagnosis, treatment, and rehabilitation management of spinal diseases.

In conclusion, this study is the first to systematically analyze the potential value of IT in spinal sagittal balance assessment, particularly demonstrating its significant clinical relevance as an alternative to PT. By revealing the strong correlations between IT, PT, and PSA, we provide compelling evidence for the application of IT in the evaluation of spinal sagittal parameters. With further research and technological advancements, IT is poised to become a key parameter in the assessment of spinal sagittal balance, offering more efficient and precise solutions for both clinical practice and research.

## Data Availability

No datasets were generated or analysed during the current study.
